# Effect of sarcopenia status on higher-level functional capacity in daily living among older orthopedic outpatients in Japan

**DOI:** 10.1186/s12891-026-09834-6

**Published:** 2026-04-27

**Authors:** Yoshihito Tomita, Hiroki Nakashima, Satoshi Mizukami, Kazuhiko Arima, Yosuke Kusano, Yasuyo Abe, Mitsuo Kanagae, Kiyoshi Aoyagi

**Affiliations:** 1 Department of Physical Therapy, School of Rehabilitation, Tokyo Professional University of Health Science, 2-22-10 Shiohama, Koto, Tokyo, 135-0043 Japan; 2 Department of Public Health, Nagasaki University Graduate School of Biomedical Sciences, Nagasaki, Japan; 3 Department of Nursing, Nishi-Kyusyu University, Ogi, Japan; 4 Department of Health and Nutrition Science, Nishi-Kyusyu University, Kanzaki, Japan; 5 Department of Rehabilitation, Nishi-Isahaya Hospital, Isahaya, Japan

**Keywords:** Disability, Older, Orthopedic outpatients, Sarcopenia, Social role

## Abstract

**Background:**

A higher risk of disability has been reported in sarcopenia and severe sarcopenia patient groups than in the non-sarcopenia group. Here, we showed an independent association between sarcopenia and disability among older orthopedic outpatients in Japan.

**Methods:**

The sample included 103 older outpatients aged ≥ 65 years with orthopedic diseases. Body mass index (BMI) was calculated as weight divided by height squared (kg/m^2^). Sarcopenia was defined as low grip strength and low muscle mass. Participants were classified according to AWGS2019 criteria: no sarcopenia (normal muscle strength, mass, and physical performance), sarcopenia (low muscle strength plus low muscle mass), or severe sarcopenia (low muscle strength plus low muscle mass plus low physical performance). Functional capacity was evaluated using the Tokyo Metropolitan Institute of Gerontology Index of Competence (TMIG-IC), a multidimensional 13-item scale that consists of three subscales: instrumental activities of daily living, intellectual activity, and social role. The association between sarcopenia and disability was assessed using logistic regression analysis.

**Results:**

The prevalence rates of sarcopenia and severe sarcopenia were 14.7% and 11.8%, respectively. Older age was associated with severe sarcopenia. The percentage of individuals with disabilities in social roles was significantly higher in the severe sarcopenia group than that in the non-sarcopenia group. After adjusting for age, sex, BMI, and comorbidity, severe sarcopenia was significantly associated with disability in social roles among orthopedic outpatients. The sarcopenia group showed no significant social role disabilities.

**Conclusions:**

Sarcopenia is a serious problem facing older orthopedic outpatients. Severe sarcopenia was found to be associated with disability in social roles among older orthopedic outpatients.

## Background

Rosenberg defined sarcopenia (ICD-10 code M62.84) as “age-related loss of muscle mass and function” [1, 2]. The causes of sarcopenia include aging, disuse, inadequate nutrition, endocrine and neurodegenerative diseases, and cachexia [3]. Sarcopenia increases the risk of negative health outcomes such as falls, fractures, dependency, use of hospital services, institutionalization, poor quality of life, and mortality [4].

Musculoskeletal disorders are associated with the development of sarcopenia, particularly in older orthopedic outpatients. Knee and hip pain may directly contribute to sarcopenia progression and increase the risk in older women [5]. Patients with orthopedic diseases are likely to become inactive because of pain [6]; therefore, they may be at a higher risk of developing sarcopenia. In one study, the prevalence rates of sarcopenia were 11.5% and 16.7% in older Japanese community-dwelling men and women, respectively [7]. In another study, 40% of the patients with chronic low back pain met the criteria for sarcopenia in the outpatient department of orthopedic surgery [8]. Moreover, its prevalence in patients with rheumatoid arthritis is 37.1% [9]. In orthopedic settings, musculoskeletal pain may accelerate sarcopenia through pain-related physical inactivity [6]. Understanding sarcopenia-disability relationships in this population is clinically important.

Increasing evidence suggests that sarcopenia contributes to functional disabilities in older persons [10, 11]. According to a study of patients in Western countries, in which sarcopenia was defined only by muscle mass, the likelihood of difficulty in performing instrumental activities of daily living (IADL) scale was 3.66 and 4.08 times greater in older men and women with sarcopenia, respectively, than in those without sarcopenia [12]. The disability risk has been found to be higher in the sarcopenia and severe sarcopenia groups than in the non-sarcopenia group among community-dwelling older Japanese [13]. However, the association between sarcopenia and disability has not yet been studied in older Japanese orthopedic outpatients. This study aimed to evaluate the association between sarcopenia and disability among older orthopedic outpatients in Japan.

## Methods

Participants were recruited from orthopedic patients who visited Nishi-Isahaya Hospital and voluntarily enrolled in this observational study. Between July 2016 and June 2017, we recruited patients aged ≥ 65 years attending the orthopedic outpatient clinic at Nishi-Isahaya Hospital. During this period, approximately 250 patients met the age criterion. Of these, 109 enrolled (enrollment rate: 43.6%). Written informed consent forms were available in Japanese to ensure complete understanding, and all participants provided written informed consent before examinations. This study was approved by the Ethics Committee of Tokyo Professional University of Health Sciences (project registration number: TPU-23-029).

The study sample included 109 older outpatients ≥ 65 years of age with orthopedic diseases. Our power analysis showed that the sample size was sufficient for our statistical analyzes (G*Power ver. 3.1. Test family [chi-square test], statistical test [goodness-of-fit test: contingency tables], effect size = 0.40, alpha error = 0.05, 1-beta error = 0.80, total sample size = 81). We used Cohen’s w = 0.40, representing a medium-to-large effect size [14] based on a meta-analysis in gerontology. Contingency tables were based on TMIG-IC disability status (disabled vs. not disabled) by sarcopenia category (no sarcopenia, sarcopenia, severe sarcopenia) [15]. All participants had sufficient cognitive function to complete the questionnaire and were asked if they had any comorbidities (heart disease, lung disease, stroke, or diabetes mellitus). Data on the diagnoses of musculoskeletal disorders (such as osteoarthritis, rheumatoid arthritis, and fractures) were collected. Pain sites (shoulder, elbow, wrist, finger, hip, knee, ankle, foot, upper back pain, middle back pain, and lower back pain) were recorded, but the severity of pain has not been assessed.

Height (m) and weight (kg) were measured with the participants wearing light clothing and no shoes. Body mass index (BMI) was calculated by dividing weight by height squared (kg/m^2^). Muscle mass was measured by bioelectrical impedance analysis (BIA) using InBody 430 (InBody Japan Inc., Tokyo, Japan). The BIA method requires participants to step onto a platform and remain in the standing position for approximately 30 seconds. Appendicular skeletal muscle mass index (AMI) was calculated as the sum of the muscle mass of the four limbs. Absolute appendicular muscle mass was converted to AMI, which was calculated by dividing the absolute appendicular muscle mass by the square of height in meters squared (kg/m^2^).

The grip strength of the dominant hand was measured using a Jamar hydraulic hand dynamometer (Jafayette Instrument Company, Inc., Jafayette, IN, USA). The best performance from two attempts was accepted. Walking speed was calculated as the time required for participants to complete a distance of 10-m at their usual pace (usual walking speed; average of two trials). Participants were classified as having sarcopenia based on their muscle mass, muscle strength, and physical performance. The classification was based on the recommendations of the Asian Working Group for Sarcopenia 2019 (AWGS2019) [16]. These recommendations define sarcopenia as age > 60 years, low handgrip strength (< 28 kg and 18 kg in men and women, respectively) and/or slow walking speed (< 1.0 m/s), and low AMI (< 7.0 kg/m^2^ and 5.7 kg/m^2^ in men and women, respectively). “Sarcopenia” was defined as the presence of low muscle mass, low muscle strength or poor physical performance, while “severe sarcopenia” was defined the presence of all three conditions. “Non-sarcopenia” was defined as absence of low muscle strength, low muscle mass, and poor physical performance.

**Fig. 1:**
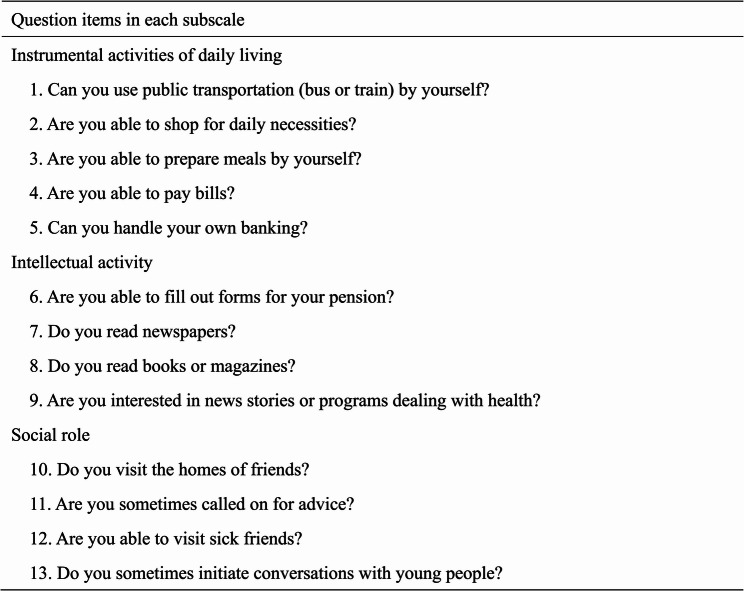
Tokyo Metropolitan Institute of Gerontology Index of Competence (TMIG-IC)

We used the Tokyo Metropolitan Institute of Gerontology Index of Competence (TMIG-IC) to measure participants’ high-functional capacity [17]. We used the TMIG-IC because it was developed for older Japanese individuals and has been widely used in the Japan community [17]. The TMIG-IC is a 13-item multidimensional scale consisting of three subscales: IADL (five items), Intellectual activity (four items), and Social roles (four items) (Fig. 1). The answer for each item was either ‘‘yes’’ (able to do, 1 point) or ‘‘no’’ (unable to do, 0 points); the maximum score was 13 points. The IADL subscale scores range from 0 to 5 points, the intellectual activity subscale scores range from 0 to 4 points, and social role subscale scores range from 0 to 4 points; higher scores reflected higher skill levels. A score of one or more below the total scores on these subscales indicated the presence of disability in the given domain. A score of 4/5 or less on the IADL or 3/4 or less on intellectual activity or social roles was considered to indicate disability in this subscale [18].

### Statistical analysis

We used the Shapiro–Wilk test for normality. Comparisons of variables among the severe sarcopenia, sarcopenia, and non-sarcopenia groups were performed using one-way ANOVA for continuous variables or Fisher’s exact test for categorical variables. The association between sarcopenia and disability was assessed using logistic regression analysis adjusted for age, sex, BMI and comorbidities. The Hosmer–Lemeshow test was used to assess the difference between the observed and predicted prevalence in the logistic regression analysis. Odds ratios (OR) and 95% confidence intervals (CIs) were calculated. Statistical significance was set at *P* < 0.05. All statistical analyses were performed using IBM SPSS Statistics, version 27 (IBM Corp., Armonk, NY, USA).

## Results

In total 109 participated. Exclusion criteria were participants with pacemaker implantation (*n* = 1) and missing values for diagnosing sarcopenia (*n* = 5). Finaly, 103 participants in the analysis. The prevalence rates of musculoskeletal diseases among the participants was as follows: osteoarthritis (*n* = 47, 46.1%), rheumatoid arthritis (*n* = 4, 3.9%), fracture (*n* = 44, 43.1%), and other (*n* = 7, 6.9%). All participants experienced at least musculoskeletal pain in at least one site (Table 1). The common sites of pain were shoulder (*n* = 71, 68.9%), elbow (*n* = 13, 12.6%), wrist (*n* = 12, 11.7%), finger (*n* = 11, 10.7%), hip (*n* = 17, 16.5%), knee (*n* = 52, 50.5%), ankle (*n* = 16, 15.5%), foot (*n* = 11, 10.7%), upper back pain (*n* = 26, 25.2%), mid back pain (*n* = 13, 12.6%), and low back pain (*n* = 67, 65.0%).

**Table 1:** Sites of pain (*N* = 103)

Pain site	*n* (%)
Shoulder	71 (68.9)
Elbow	13 (12.6)
Wrist	12 (11.7)
Finger	11 (10.7)
Hip	17 (16.5)
Knee	52 (50.5)
Ankle	16(15.5)
Foot	11 (10.7)
Upper back	26 (25.2)
Mid back	13 (12.6)
Low back	67 (65.0)

Table 2 shows the characteristics of participants. The mean ages and BMI were 76.8 years and 23.5 kg/m^2^, respectively. The percentages of IADL disability, intellectual activity, and social roles were 11.7%, 33.0%, and 41.7%, respectively. The prevalence rates of sarcopenia and severe sarcopenia were 14.7% (*n* = 15) and 11.8% (*n* = 12), respectively (Table 3). The participants with severe sarcopenia had significantly lower BMI (*P* = 0.001), AMI (*P* < 0.001), grip strength (*P* < 0.001), and walking speed (*P* < 0.001) than those with non-sarcopenia. Advanced age (*P* = 0.036) was associated with severe sarcopenia. The proportions of disabilities in social roles were significantly higher in the severe sarcopenia group than that in the non-sarcopenia group (*P* = 0.043).

**Table 2:** Characteristics of the participants (*N* = 103)

	Mean	SD
Age, years	76.8	7.1
Body mass index, kg/m^2^	23.5	3.9
Appendicular muscle mass index, kg/m^2^	6.1	0.9
Grip strength, kg	22.5	6.5
Usual walking speed, m/s	1.1	0.2

	Number	%
Sex, women	83	80.6
Comorbidity^a^, yes	78	75.7
Instrumental ADL^b^ disability^c^, yes	12	11.7
Intellectual activity disability^d^, yes	34	33.0
Social role disability^e^, yes	43	41.7

^a﻿^Presence of heart disease, lung disease, stroke, or diabetes mellitus

^b﻿^ADL: Activities of daily living

^c﻿^A score of 4 or less out of 5 for Instrumental ADL^b^ was considered to indicate the presence of a disability

^d﻿^A score of 3 or less out of 4 for intellectual activity was considered to indicate the presence of a disability

^e﻿^A score of 3 or less out of 4 for social role was considered to indicate the presence of a disability

**Table 3:** Comparison of variables among severe sarcopenia, sarcopenia, and non-sarcopenia group (*N* = 103)

	Non-sarcopenia (*n* = 76)		Sarcopenia (*n* = 15)		Severe sarcopenia (*n* = 12)		*P* value
	Mean	SD^f^	Mean	SD^f^	Mean	SD^f^	
Age, years	76.1	6.6	76.8	6.4	81.7	8.9	0.036
Body mass index, kg/m^2^	24.3	3.9	21.1	2.0	21.1	2.0	0.001
Appendicular muscle mass index, kg/m^2^	6.4	0.9	5.4	0.6	5.4	0.6	< 0.001
Grip strength, kg	24.0	6.5	19.2	4.1	17.4	5.1	< 0.001
Usual walking speed, m/s	1.1	0.2	0.8	0.1	0.8	0.1	< 0.001
	Number	%	Number	%	Number	%	
Comorbidity^a^, yes	56	73.7	14	93.3	8	66.7	0.198
Instrumental ADL^b^ disability^c^, yes	8	10.5	1	6.7	3	25.0	0.282
Intellectual activity disability^d^, yes	26	34.2	2	13.3	6	50.0	0.120
Social role disability^e^, yes	29	38.2	5	33.3	9	75.0	0.043

^a﻿^Presence of heart disease, lung disease, stroke, or diabetes mellitus

^b﻿^ADL: Activities of daily living

^c﻿^A score of 4 or less out of 5 for Instrumental ADL^b^ was considered to indicate the presence of a disability

^d﻿^A score of 3 or less out of 4 for intellectual activity was considered to indicate the presence of a disability

^e﻿^A score of 3 or less out of 4 for social role was considered to indicate the presence of a disability

^f﻿^SD: Standard deviation, one-way ANOVA, Fisher’s exact test

After adjustments for age, sex, and comorbidity, severe sarcopenia group was found to be significantly associated with disability for social role (OR: 5.19, 95% CI: 1.21–22.32) in orthopedic outpatients (Table 4).

**Table 4:** Association between sarcopenia status and social role disability (*N* = 103)

	Unit	Sarcopenia and severe sarcopenia (*n* = 27) versus non-sarcopenia (*n* = 76)	Sarcopenia (*n* = 15) versus non-sarcopenia (*n* = 76)	Severe sarcopenia (*n* = 12) versus non-sarcopenia (*n* = 76)
		Odds(95% Confidence interval)	Odds(95% Confidence interval)	Odds(95% Confidence interval)
Instrumental ADL^b^ disability^c^	yes	1.13 (0.28–4.51)	0.63 (0.07–5.84)	1.74 (0.32–9.36)
Age	5 years	1.31 (0.94–1.84)	1.01 (0.64–1.57)	1.79 (1.09–2.94)*
Sex	Women	0.81 (0.25–2.59)	0.71 (0.17–3.05)	1.12 (0.19–5.41)
Comorbidity^a^	yes	1.45 (0.46–4.58)	5.11 (0.60–43.64)	0.57 (0.13–2.44)
Intellectual activity disability^d^	yes	0.83 (0.30–2.26)	0.37 (0.07–1.82)	1.68 (0.44–6.45)
Age	5 years	1.33 (0.96–1.85)	0.96 (0.60–1.51)	1.81 (1.11–2.94)*
Sex	Women	0.79 (0.26–2.46)	0.78 (0.18–3.34)	0.88 (0.18–4.34)
Comorbidity^a^	yes	1.36 (0.42–4.43)	4.32 (0.49–38.16)	0.64 (0.14–2.92)
Social role disability^e^	yes	1.76 (0.70–4.38)	0.88 (0.27–2.93)	5.19 (1.21–22.32)*
Age	5 years	1.31 (0.95–1.83)	1.00 (0.63–1.56)	1.92 (1.14–3.24)*
Sex	Women	0.85 (0.27–2.67)	0.74 (0.17–3.13)	1.03 (0.19–5.14)
Comorbidity^a^	yes	1.52 (0.48–4.86)	5.16 (0.61–43.89)	0.55 (0.12–2.56)

Logistic regression analysis

^a﻿^Presence of heart disease, lung disease, stroke, or diabetes mellitus

^b﻿^ADL: Activities of daily living

^c﻿^A score of 4 or less out of 5 for Instrumental ADL^b^ was considered to indicate the presence of a disability

^d﻿^A score of 3 or less out of 4 for intellectual activity was considered to indicate the presence of a disability

^e﻿^A score of 3 or less out of 4 for social role was considered to indicate the presence of a disability

^*^P value < 0.05

## Discussion

### Prevalence of sarcopenia

The prevalence of sarcopenia (14.7%) and severe sarcopenia (11.8%) in our orthopedic outpatients was higher than in Japanese community-dwelling older adults (8–12%) [19]. Among community-dwelling older adults aged ≥ 60 years in Asia, the prevalence of sarcopenia is approximately 16–17%, while severe sarcopenia accounts for about 4–5% [20]. The higher prevalence in orthopedic settings may reflect the bidirectional relationship between musculoskeletal disorders and sarcopenia: chronic pain reduces activity, accelerating muscle loss [6], while sarcopenia increases fall and injury risk [21]. International studies using comparable criteria report community prevalence of 12.9% [22] and clinical population prevalence of 23–24% [23]. Sarcopenia and severe sarcopenia are serious problems among facing orthopedic outpatients.

### Severe sarcopenia and social role

Our study showed that severe sarcopenia was associated with disability in social roles among older orthopedic outpatients. Previous reports have suggested that sarcopenia is significantly associated with IADL and social role disability [24]. Thus, not only sarcopenia but also severe sarcopenia with low muscle mass, low muscle strength, and low physical performance may be associated with impairment of social roles in older adults. A longitudinal study showed that severe sarcopenia, involving low muscle mass and low physical performance, increased the risk of disability in older adults compared with that with low muscle mass alone [13]. Conversely, maintaining good social relationships may contribute to the prevention of severe sarcopenia; further longitudinal research is needed to clarify the causality between severe sarcopenia and social role disability.

### Sarcopenia and social role

In the Kashiwa cohort, social role was not independently associated with overall sarcopenia after multivariable adjustment, despite its associations with gait speed and more advanced disease stages reported in previous studies [25]. This discrepancy may reflect the indirect nature of social role, which primarily influences sarcopenia through proximal mediators such as physical activity, nutrition, psychological status, and oral function [25]. Once these factors are accounted for, the direct association with the presence of sarcopenia may be attenuated, particularly given the relatively low prevalence of sarcopenia and limited statistical power. Consequently, social role may contribute more to disease progression and severity rather than to the initial presence of sarcopenia.

### Sarcopenia and intellectual activity disability in IADL

The prevalence of mild cognitive impairment is relatively high in patients with sarcopenia, and may be a risk factor for sarcopenia [26]. Sarcopenia is significantly associated with IADL disability in older Japanese people [24]. In an 8-year follow-up study of older Japanese people, disability in social roles was the most prevalent disability, followed by disabilities in intellectual activity and IADL [27]. Similarly, our study showed that among men and women with sarcopenia or severe sarcopenia, the percentage of disability in social roles was the highest, followed by disabilities in intellectual activity and IADL. However, this study showed no significant differences between sarcopenia and disabilities in intellectual activity and IADL.

### Limitations

This study has several limitations. First, our modest sample size resulted in limited events-per-variable (EPV) ratios in multivariable analyses. While recent simulation studies suggest acceptable performance with EPV ratios of 5–9 [28, 29], our findings should be interpreted cautiously and require validation in larger cohorts. Second, because this study had a cross-sectional design, we could not establish a causal relationship between sarcopenia and disability. Therefore, longitudinal studies are needed to determine causality. Third, outcomes were collected from older Japanese orthopedic outpatients. Therefore, these results cannot be extrapolated to other ethnic groups or community-dwelling populations. Fourth, although all participants experienced musculoskeletal pain, exploratory analyses examining interaction effects between sarcopenia status and the number of pain sites on functional capacity did not show statistically significant interactions. However, our limited sample size may have restricted statistical power to detect such effects, and future studies with larger samples are needed to clarify this relationship. Fifth, it may be difficult to assess the severity of comorbidities in older patients. Therefore, severity was not assessed. Moreover, information on medications or pain severity was not collected. These limitations may have contributed to the underestimation of the associations reported herein. Finally, the possibility of selection bias in this single-hospital study cannot be ignored. Further multicenter studies are needed.

## Conclusion

Sarcopenia is a serious problem in older orthopedic outpatients. Our study showed that severe sarcopenia was associated with disability in social roles among older orthopedic outpatients in Japan.

## Data Availability

The datasets used and/or analyzed during the current study are available from the corresponding author on reasonable request.

## Abbreviations

IADL: Instrumental activities of daily living
BMI: Body mass index
BIA: Bioelectrical impedance analysis
AMI: Appendicular skeletal muscle mass index
AWGS: Asian Working Group for Sarcopenia
TMIG-IC: Tokyo Metropolitan Institute of Gerontology Index of Competence
